# Preventing zoonotic diseases in immunocompromised persons: the role of physicians and veterinarians.

**DOI:** 10.3201/eid0501.990121

**Published:** 1999

**Authors:** S Grant, C W Olsen

**Affiliations:** University of Wisconsin, Madison, Wisconsin, USA.

## Abstract

We surveyed physicians and veterinarians in Wisconsin about the risk for and prevention of zoonotic diseases in immunocompromised persons. We found that physicians and veterinarians hold significantly different views about the risks posed by certain infectious agents and species of animals and communicate very little about zoonotic issues; moreover, physicians believe that veterinarians should be involved in many aspects of zoonotic disease prevention, including patient education.


### Preventing Zoonotic Diseases in Immunocompromised Persons: The Role of Physicians and Veterinarians

**Sara Grant and Christopher W. Olsen**
University of Wisconsin, Madison, Wisconsin, USA

We surveyed physicians and veterinarians in Wisconsin about the risk for and prevention of zoonotic diseases in immunocompromised persons. We found that physicians and veterinarians hold significantly different views about the risks posed by certain infectious agents and species of animals and communicate very little about zoonotic issues; moreover, physicians believe that veterinarians should be involved in many aspects of zoonotic disease prevention, including patient education.

The bond between humans and animals has been recognized for many years, and pet ownership has been associated with both emotional and health benefits (1-4). However, pet ownership may also pose health risks through the zoonotic transmission of infectious diseases, especially in the immunocompromised (5). Animal-associated pathogens of concern to immunocompromised persons include *Toxoplasma gondii*, *Cryptosporidium* spp., *Salmonella* spp., *Campylobacter* spp., *Giardia lamblia*, *Rhodococcus equi*, *Bartonella* spp., *Mycobacterium marinum*, *Bordetella bronchiseptica, Chlamydia psittaci,* and zoophilic dermatophytes (2,6). However, with the exception of *Bartonella henselae* and zoophilic dermatophytes, infections in humans are more commonly acquired from sources other than pets, and the infectious disease risk from owning pets is considered low (2,7). Nonetheless, HIV-infected persons may still be advised not to own pets (8).

Since human medicine often does not delve deeply into the role of animals in the transmission of zoonotic agents (7,9) and veterinary medicine does not cover the clinical aspects of human disease, zoonotic disease control requires involvement of both physicians and veterinarians. We examined how frequently physicians and veterinarians encounter zoonotic diseases, what role physicians think veterinarians should play in zoonotic disease prevention, how often physicians and veterinarians communicate about zoonoses issues, and what physicians and veterinarians perceive as the disease risk of immunocompromised persons from pets.

Our sample populations were drawn from membership lists of the Wisconsin Veterinary Medical Association (WVMA) and the State Medical Society of Wisconsin (excluding retired practitioners). Veterinarians (n = 526) were chosen by a systematic sampling of every third name on the WVMA membership list. Since veterinarians in all types of practice may encounter zoonotic problems, sampling was not stratified by specialty. Physicians (n=698) were chosen by specialty most likely to involve both zoonotic diseases and immunocompromised patients (all physicians who listed infectious disease [n = 38] or hematology/oncology [n = 103] as specialties), as well as randomly selected cohorts of pediatricians (n = 100), and general internal medicine physicians (n = 500). Duplicate names were removed.

Each participant was mailed a cover letter; a number-coded, postage-paid return envelope; and a physician- or veterinarian-specific survey. Nonresponders received a second survey 3 weeks after the first. For questions with a response scale of 1 to 5, the sample size was large enough for a 2-sample Z-test to statistically compare mean responses between physicians and veterinarians. Additionally, responses were analyzed by veterinary practice type and physician

Address for correspondence: Christopher W. Olsen, Department of Pathobiological Sciences, School of Veterinary Medicine, University of Wisconsin, 2015 Linden Drive West, Madison, WI 53706, USA; fax: 608-263-0438; e-mail: olsenc@svm.vetmed.wisc.edu.

specialty. Statistically significant differences in responses by specialty are noted in the text. A 2-sample Z-test employing the standard large sample approximation to binomial data was used to compare the proportions of responses by physicians and veterinarians to the question about zoonotic pathogens of concern to immunocompromised patients.

Surveys were completed by 327 veterinarians and 322 physicians (overall response rate of 53%). Responses from veterinarians were as follows: 142 (43%) small-animal practice, 65 (20%) large-animal practice, 98 (30%) mixed-animal practice, and 22 (7%) exotic-animal practice. The distribution of respondents by practice type is very similar to the distribution by practice type across the State of Wisconsin (46% small animal, 19% large animal, 35% mixed, and 0.3% exotic [data courtesy of M. Mardock, Wisconsin Veterinary Medical Association]), except for an overrepresentation of exotic-animal practitioners. Nationally, 8% of veterinarians are in large animal practice exclusively, 29% in mixed practice, 58% in small animal practice and 5% "other" (data from the Veterinary Economics Statistics Brochure of the AVMA, September 1998). The distribution of responses from physicians included 24 (7%) in infectious diseases, 48 (15%) in hematology/oncology, 53 (16%) in pediatrics and 197 (61%) in general internal medicine (including 16 who specifically categorized themselves as pulmonologists and 11 as rheumatologists). Among our random selection of pediatricians and general internists, the respondent ratio of 3.7 internists for every 1 pediatrician is slightly higher than the statewide ratio of 2.5 (Wisconsin physician data courtesy of M. O'Brien, State Medical Society of Wisconsin) and the national ratio of 2.0 (10).

The survey results indicate that veterinarians (Table 1) encounter zoonotic diseases in their practices or discuss them with their clients more frequently ($p < 0.00001$) than physicians (Table 2). Among veterinarians, small-animal practitioners encounter zoonoses more frequently than veterinarians as a whole (mean = 2.80, $p = 0.05$), and large-animal veterinarians less frequently (mean = 3.41, $p = 0.001$). Among physicians, infectious disease specialists encoun-

Table 1. Survey of veterinarians

| Questions | Responses |
|---|---|
| How often do you encounter or discuss zoonotic diseases in your patient population? 1=Several times/day; 2=Daily; 3=Weekly; 4=Occasionally; 5=Never | $\bar{X}$ = 3.02[a] (±0.05)[b] |
| How often do physicians contact you for advice on the animal aspects of transmission and risks of zoonotic diseases? 1=Several times/week; 2=Several times/month; 3=Several times/year; 4=Rarely; 5=Never | $\bar{X}$ = 4.30 (±0.04) |
| How often do you contact physicians regarding a zoonotic disease? 1=Several times/week; 2=Several times/month; 3=Several times/year; 4=Rarely; 5=Never | $\bar{X}$ = 4.21 (±0.04) |
| If you know that a client is immunocompromised, do you offer consultation on zoonotic disease prevention? | |
| - Yes | n=96[c] |
| - No | n=9 |
| - The situation has never arisen | n=205 |
| How much risk to immunocompromised patients is associated with owning or having contact with the following animals? 1=Highest risk to 5=Lowest risk | |
| - Reptile | $\bar{X}$ = 2.28 (±0.09) |
| - Bird | $\bar{X}$ = 2.49 (±0.07) |
| - Kitten (<6 months of age) | $\bar{X}$ = 2.81 (±0.07) |
| - Puppy (<6 months of age) | $\bar{X}$ = 3.02 (±0.07) |
| - Farm animals | $\bar{X}$ = 3.05 (±0.07) |
| - Cat | $\bar{X}$ = 3.28 (±0.06) |
| - Dog | $\bar{X}$ = 3.86 (±0.06) |

[a]Mean of all respondents.
[b]Standard error of the mean.
[c]Absolute number of veterinarians answering "yes", "no" or "the situation has never arisen".

Table 2. Survey of physicians

| Questions | Responses |
|---|---|
| How often do you encounter or discuss zoonotic diseases in your patient population? | |
| 1=Several times/day; 2=Daily; 3=Weekly; 4=Occasionally; 5=Never | $\bar{x}$ = 4.16[a] (±0.03)[b] |
| How comfortable do you feel in advising patients specifically on the animal aspects of transmission and the risks for zoonotic diseases? | |
| 1=Very comfortable to 5=Not comfortable | $\bar{x}$ = 3.69 (±0.05) |
| Should veterinarians be involved in advising clients about the potential for zoonotic disease? | |
| 1=Veterinarian should have primary responsibility; | $\bar{x}$ = 2.77 (±0.05) |
| 3=Responsibility should be equal; 5= Physician should have primary responsibility | |
| How involved should veterinarians be in the following areas in reducing transmission of zoonotic disease agents to immunocompromised patients, providing that client confidentiality is maintained? | |
| 1=Very involved to 5=Not involved | |
| - General maintenance of animal health | $\bar{x}$ = 1.62 (±0.06) |
| - Additional zoonotic disease screening of animals | $\bar{x}$ = 1.78 (±0.06) |
| - Zoonoses education for patients | $\bar{x}$ = 2.08 (±0.06) |
| - Consultation for physicians | $\bar{x}$ = 2.12 (±0.06) |
| How often do veterinarians contact you regarding zoonotic diseases? | |
| 1=Several times/week; 2=Several times/month; 3=Several times/year; 4=Rarely; 5=Never | $\bar{x}$ = 4.74 (±0.03) |
| How often do you contact veterinarians for advice on the animal aspects of transmission and risks of zoonotic diseases? | |
| 1=Several times/week; 2=Several times/month; 3=Several times/year; 4=Rarely; 5=Never | $\bar{x}$ = 4.55 (±0.03) |
| How much risk to immunocompromised patients is associated with owning or having contact with the following animals? | |
| 1=Highest risk to 5=Lowest risk | |
| - Bird | $\bar{x}$ = 2.37 (±0.07) |
| - Kitten (<6 months of age) | $\bar{x}$ = 2.47 (±0.08) |
| - Cat | $\bar{x}$ = 2.58 (±0.07) |
| - Reptile | $\bar{x}$ = 2.64 (±0.09) |
| - Farm animals | $\bar{x}$ = 2.94 (±0.08) |
| - Puppy (<6 months of age) | $\bar{x}$ = 3.28 (±0.08) |
| - Dog | $\bar{x}$ = 3.69 (±0.06) |

[a]Mean of all respondents.
[b]± Standard error of the mean.

ter zoonoses more frequently than the overall population of physician respondents (mean = 3.44, p = 0.001), but these specialists still encounter zoonoses problems less frequently than veterinarians (p = 0.05).

When physicians were asked (on a scale of 1 to 5 with 1 = very comfortable and 5 = not comfortable) how comfortable they felt about advising patients on the role of animals in the transmission of zoonotic agents and associated risks, with the exception of infectious disease specialists (whose mean comfort level = 1.92 was significantly [p ≤ 0.0001] better than that of the overall population of physicians), they responded that they were not very comfortable in this role (mean = 3.69, Table 2); moreover, physicians indicated that veterinarians should play an equal or greater role in advising patients about zoonotic diseases (Table 2). In particular, they suggested that veterinarians should be involved not only in controlling zoonotic disease pathogens in animals, but also in providing information for patients and physicians (Table 2). However, the survey demonstrated a nearly complete lack of communication between physicians and veterinarians about zoonotic disease issues (Tables 1,2). In addition, patients themselves do not appear to view veterinarians as a source of zoonotic disease information. Of 310 veterinarians, 96 indicated that they offer special consultation about additional steps for zoonotic disease prevention if they are aware of the fact that a client is immunocompromised; however, for 205 of 310 respondents, the client's health was never discussed (Table 1).

In the second portion of the survey, we examined the views of physicians and veterinarians on the possible disease risks (from specific animals or pathogens) to immunocompromised persons. Various animals were ranked on a risk scale of 1 to 5 (1 = highest risk to 5 = lowest risk, with an option to respond "unsure"). Veterinarians assigned a higher risk than physicians to reptiles ($p = 0.004$) and puppies ($p = 0.01$); physicians assigned a higher risk than veterinarians to cats ($p \leq 0.00001$) and kittens ($p = 0.001$) (Tables 1,2). Physicians and veterinarians were also asked to list the two zoonotic pathogens of greatest concern for immunocompromised persons (Table 3). The two most frequently named pathogens were *Salmonella* spp. and *Toxoplasma gondii.* Within this ranking, *Salmonella* spp. were listed more frequently ($p = 0.001$) by veterinarians than physicians, and this concern may explain why veterinarians thought that reptiles pose the greatest risk to the immunocompromised (Table 1). (Because of the high prevalence in reptiles of *Salmonella* infection, immunocompromised persons are advised not to own or handle reptiles [2,11]). In contrast, *T. gondii* was listed as a potential disease risk more often by physicians ($p = 0.001$), which is consistent with physician's concern about immunocompromised persons owning kittens and cats as pets (Table 2).

In summary, our survey results indicate that physicians and veterinarians hold very different views about the disease risks from certain animals and infectious agents and communicate very little about zoonotic disease prevention. The perceived risks posed by specific pathogens raise some questions. First, for both *Salmonella* spp. and *T. gondii*, contact with pets is not the only, or even the most important, source of infection for humans. Contaminated foods are the most common vehicle of *Salmonella* spp. infection (12); undercooked meat is also a common vehicle of *T. gondii* infection. Up to 25% of lamb and pork samples contain *Toxoplasma* tissue cysts (13). Therefore, although cats are the definitive hosts for *T. gondii*, cat ownership is not associated with an increase in *Toxoplasma* seroconversion among HIV-infected persons (14). Secondly, a number of the infectious disease agents (e.g., *Borrelia burgdorferi*, *Histoplasma capsulatum*, *Blastomyces dermatitidis*, and *Pneumocystis carinii*) listed as zoonotic disease risks by both physicians and veterinarians are not truly zoonotic, but rather shared infections. Both animals and humans are infected, but animals are not the direct vehicles of infection for humans. In addition, cytomegaloviruses of humans and animals are not infectious across species. Finally, it is surprising that infection with *B. henselae,* the causative agent of cat scratch disease, which also causes bacillary angiomatosis, peliosis hepatis, and other conditions in immunocompromised persons, was listed relatively infrequently by both physicians and veterinarians (Table 3). Exposure to kittens has been clearly implicated as a significant risk factor in the epidemiology of *B. henselae* (15). Both physicians and veterinarians need to recognize the role of this pathogen in the zoonotic infection of immunocompromised persons and the role of cats in its transmission.

Table 3. Responses of physicians and veterinarians when asked to "List the two zoonotic pathogens you believe should be of greatest concern for immunocompromised individuals"

| Pathogen | Physicians | Veterinarians |
|---|---|---|
| *Toxoplasma gondii* | n[a] = 144 | n[b] = 74[c] |
| *Salmonella* spp. | n = 61 | n = 111[c] |
| *Cryptosporidium parvum* | n = 54 | n = 86 |
| *Mycobacterium* spp. | n = 29 | n = 18 |
| *Chlamydia psittaci* | n = 24 | n = 31 |
| *Bartonella* spp. | n = 15 | n = 10 |
| *Histoplasma capsulatum* | n = 13 | n = 1[c] |
| *Giardia lamblia* | n = 12 | n = 14 |
| *Pasteurella* spp. | n = 9 | n = 6 |
| *Borrelia burgdorferi* | n = 8 | n = 6 |
| *Pneumocystis carinii* | n = 8 | n = 2 |
| Cytomegalovirus | n = 8 | n = 0[c] |
| *Blastomyces dermatitidis* | n = 7 | n = 9 |
| Rabies virus | n = 6 | n = 2 |
| *Campylobacter* spp. | n = 5 | n = 10 |
| *Escherichia coli* | n = 2 | n = 11[d] |
| *Streptococcus* spp. | n = 1 | n = 9[d] |
| Dermatophytes | n = 1 | n = 24[c] |
| "Unsure" | n = 29 | n = 9 |
| Total number of participants responding to this question | n = 259 | n = 271 |

[a]Total number of times each agent was listed by physicians.
[b]Total number of times each agent was listed by veterinarians.
[c,d]The number of times these organisms were listed by physicians and veterinarians were significantly different. ([c]$p \leq 0.001$; [d]$p=0.02$ ).

Our finding that 205 of 310 veterinarians never knew a client's immunocompromised condition is consistent with a previous study in which only 21% of HIV patients felt most comfortable in asking their veterinarian about the health risks of pet ownership (16). Through approaches such as small signs in exam rooms, zoonotic disease brochures in reception areas, comments in practice newsletters, and affiliation with support groups in the community, veterinarians can encourage immunocompromised persons to avail themselves of the diagnostic and preventive measures that can be provided for zoonotic agents.

Our results suggest that communication between physicians and veterinarians about zoonotic diseases is largely absent. Enhancing such communication could help prevent transmission of zoonotic agents. In addition to directly contacting veterinary practitioners in their community, physicians can also contact their state health departments for information, since some health departments have public health veterinarians on staff. Links between the professions on a broader scale (e.g., through combined veterinary/medical student training and continuing education) to foster a broader consensus about zoonotic disease risks and prevention should also be encouraged.

### Acknowledgments

We thank Linda Sullivan, George Mejicano, Ken Felz, Barbara Burell, and Winifred Grant for assistance in designing or distributing the surveys and Chet Thomas, Rick Nordheim, Brian Aldridge, Sandra Martin, and Tom Tabone for assisting in data analysis.

The Geraldine R. Dodge Foundation and the Bernice Barbour Foundation supported this research.

Dr. Grant is a staff veterinarian at the New Haven Central Hospital for Veterinary Medicine in New Haven, Connecticut. She conducted this research during her studies at the University of Wisconsin School of Veterinary Medicine.

Dr. Olsen is an assistant professor of public health at the University of Wisconsin School of Veterinary Medicine. His research interests include DNA vaccine development and immunity to influenza viruses in horses and pigs, as well as the molecular epidemiology of swine influenza viruses as zoonotic agents.

### References

1. Fitzgerald FT. The therapeutic value of pets. West J Med 1986;144:103-5.
2. Angulo FJ, Glaser CA, Juranek DD, Lappin MR, Regnery RL. Caring for pets of immunocompromised persons. J Am Vet Med Assoc 1994;205:1711-8.
3. Burton BJ. Pets and PWAs: claims of health risk exaggerated. AIDS Patient Care 1989;3:34-7.
4. Beck AM, Meyers NM. Health enhancement and companion animal ownership. Annu Rev Public Health 1996;17:247-57.
5. AIDS patients can acquire some infections from animals [News]. J Am Vet Med Assoc 1990;197:1268-9.
6. Greene CE. Pet ownership for immunocompromised people. In: Bonagura JD, editor. Kirk's current veterinary therapy XII. Philadelphia: WB Saunders Company; 1995. p. 271-6.
7. Glaser CA, Angulo FJ, Rooney JA. Animal-associated opportunistic infections among persons infected with the human immunodeficiency virus. Clin Infect Dis 1994;18:14-24.
8. Spencer L. Study explores health risks and the human animal bond. J Am Vet Med Assoc 1992;201:1669.
9. Tan JS. Human zoonotic infections transmitted by dogs and cats. Arch Intern Med 1997;157:1933-43.
10. Seidman B, Pasko T, editors. Physician characteristics and distribution. Chicago: American Medical Association; 1998. p 57-60.
11. Centers for Disease Control and Prevention. 1997 USPHS/IDSA guidelines for the prevention of opportunistic infections in persons infected with human immunodeficiency virus. MMWR Morb Mortal Wkly Rep 1997;46:4-44.
12. Angulo FJ, Swerdlow DL. Bacterial enteric infections in persons infected with human immunodeficiency virus. Clin Infect Dis 1995;21:S84-93.
13. Dubey JP. A review of toxoplasmosis in pigs. Vet Parasitol 1986;19:181-223.
14. Wallace MR, Rosetti RJ, Olson PE. Cats and toxoplasmosis risk in HIV-infected adults. JAMA 1993;269:76-7.
15. Zangwill KM, Hamilton DH, Perkins BA, Regnery RL, Plikaytis BD, Hadler JL, et al. Cat scratch disease in Connecticut—epidemiology, risk factors, and evaluation of a new diagnostic test. N Engl J Med 1993;329:8-13.
16. St Pierre LA, Kreisle RA, Beck AM. Role of veterinarians in educating immunocompromised clients on the risks and benefits of pet ownership. In: Proceedings of the Geraldine R. Dodge Foundation Gathering and Reports of 1996 Veterinary Student Fellows; 1996 Sep 27-29; Ithaca, New York. Morristown (NJ): The Foundation; 1996.

